# Interleukin-33 and Inflammatory Bowel Diseases: Lessons from Human Studies

**DOI:** 10.1155/2014/423957

**Published:** 2014-02-20

**Authors:** Tiago Nunes, Claudio Bernardazzi, Heitor S. de Souza

**Affiliations:** ^1^Nutrition and Immunology Chair, Research Center for Nutrition and Food Sciences (ZIEL), Technische Universität München, Gregor-Mendel-Straße 2, 85354 Freising-Weihenstephan, Germany; ^2^Serviço de Gastroenterologia & Laboratório Multidisciplinar de Pesquisa, Hospital Universitario, Universidade Federal do Rio de Janeiro, Rua Prof. Rodolpho Paulo Rocco 255, Ilha do Fundão, 21941-913 Rio de Janeiro, RJ, Brazil; ^3^D'Or Institute for Research and Education, Rua Diniz Cordeiro 30, Botafogo, 22281-100 Rio de Janeiro, RJ, Brazil

## Abstract

Interleukin- (IL-) 33 is a widely expressed cytokine present in different cell types, such as epithelial, mesenchymal, and inflammatory cells, supporting a predominant role in innate immunity. IL-33 can function as a proinflammatory cytokine inducing Th2 type of immune response being involved with the defense against parasitic infections of the gastrointestinal tract. In addition, it has been proposed that IL-33 can act as a signaling molecule alerting the immune system of danger or tissue damage. Recently, in the intestinal mucosa, overexpression of IL-33 has been reported in samples from patients with inflammatory bowel diseases (IBD). This review highlights the available data regarding IL-33 in human IBD and discusses emerging roles for IL-33 as a key modulator of intestinal inflammation.

## 1. Introduction

Inflammatory bowel diseases (IBD) as ulcerative colitis (UC) and Crohn's disease (CD) are complex immune-mediated illnesses that affect genetically susceptible individuals after exposure to certain environmental factors [1]. In IBD, an inappropriate innate immune response triggered by antigens of the intestinal microbiota leads to chronic intestinal inflammation and tissue damage [1–3]. This complex genetic-environment interaction has been a matter of intense research in the past two decades, providing novel interesting insights into the IBD pathogenesis. A variety of immunological changes have been shown to occur in IBD contributing to the development of mucosal immune abnormalities, including the presence of altered subsets of inflammatory cells and the chronic activation of proinflammatory pathways [2, 4]. In this multifaceted context, interleukin- (IL-) 33 emerges as a potential novel target in IBD.

This review aims to examine the current evidence regarding the association between IL-33 and IBD in human studies. Even though some data from animal models for intestinal inflammation are briefly discussed, this is not the main focus of this review. For IBD animal studies on IL-33, a very recent review by Theresa Pizarro's group published in Mediators of Inflammation has extensively covered the topic [5].

### 1.1. IBD as an Immune-Mediated Disease

Even though UC and CD share a number of genetic and phenotypic features, these conditions are two distinct entities with regard to their underlying immunological mechanisms. On the one hand, CD is characterized by a predominant T-helper cells type-1 (Th1) immune response, dominated by the production of proinflammatory cytokines like IFN-*γ*, IL-2, and TNF-*α* [6, 7]. On the other hand, UC is an immune-mediated disease due to abnormal T-helper cells type-2 (Th2) response, characterized by an enhanced production of IL-13, IL-10, IL-6, and IL-5 [8]. In addition to these major immune responses associated with CD and UC, T-helper 17 (Th17) lymphocytes represent a third T-helper linage of CD4+ effectors in the immune system, which has also been linked to IBD [9, 10]. These Th17 cells overexpress transcription factors retinoic acid related orphan receptor (ROR)-*γ*t and ROR*α* and produce IL-17, IL-21, IL-22, and IL-26, being negatively regulated by IFN-*γ* [11, 12]. Currently, though there is a clear role for the Th17 axis in several immune-mediated diseases as rheumatoid arthritis, multiple sclerosis, psoriasis, and lupus, data are less convincing and homogeneous with respect to IBD [13].

**Figure 1:**
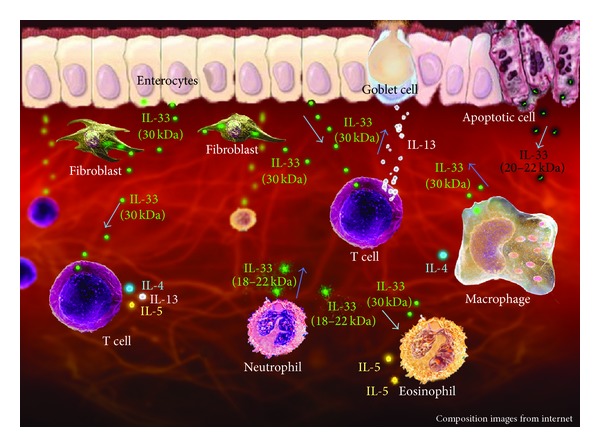
Representation of IL-33 function in the gastrointestinal mucosa. Full-length IL-33 (30 kDa) is released by a wide range of different cell types, represented here by enterocytes, fibroblasts, and macrophages. IL-33 interacts with lamina propria T cells and determines the production of IL-4, IL-5, and IL-13. IL-13 enhances mucus production by goblet cells, while IL-5 activates eosinophils and B cells, and IL-4 induces Th2 polarization. IL-33 can also activate eosinophils and macrophages, further contributing to a Th2 response in the lamina propria. Neutrophil can release a lighter structure of IL-33 (18–22 kDa), which is known to be more active than the 30 kDa protein. During cellular apoptosis, IL-33 can be cleaved by caspases 3 and 7, generating a 20–22 kDa molecule, a potentially less active protein.

These types of immune response with their different cytokine profiles are accountable for the main physiopathological differences between UC and CD [2]. At present, much research focuses on the potential therapeutic properties of blocking cytokines associated with the development of mucosal inflammation in IBD [14]. Unfortunately, blocking cytokines has an unpredictable effect on disease outcomes, with many candidates failing to show clinical efficacy [14]. In this regard, a new potential target for pharmacological blockage is the newly discovered cytokine IL-33. IL-33 is mostly associated with Th2 immune responses, being associated with intestinal inflammation both in animal and human studies [5] (Figure 1).

### 1.2. IL-33, a Novel Cytokine

IL-33 (also known as IL-1F11 or NF-HEV) is a relatively new cytokine, which is a member of the IL-1 cytokine family that also comprises IL-1*α* and IL-18. IL-33 has been found to be secreted by a wide range of different cell types, including fibroblasts, adipocytes, smooth muscle cells, endothelial cells, macrophages, dendritic cells, and respiratory and intestinal epithelial cells [23–27]. Members of this cytokine family classically exhibit a precursor form in the cytosol that is activated by caspase-1-mediated proteolytic cleavage of the N-terminal domain. IL-33, a 30 kD protein, however, is not cleaved by caspase-1 in vivo; instead, the full-length protein is actually the bioactive form, being any posterior cleavage unnecessary for its proper function [28–31].

This cytokine, nevertheless, can be cleaved or give rise to alternative splice variants with diverse activation properties. In this regard, IL-33 can be a substrate for caspases 3 and 7, generating a lighter and less active 20–22 kD protein [30]. In contrast, IL-33 can also be enzymatically cleaved by neutrophils after exposure to elastase and cathepsin G, leading to the formation of another lighter structure with 18–22 kD which is known to be more active than the 30 kD protein [32]. Finally, IL-33 has been described to have an alternative splice variant that lacks the exons with no loss of activity compared with the complete cytokine form [33]. The existence of different IL-33 variants is allegedly part of a complex autoregulatory mechanism in which there is a fine adjustment of affinity and activity in response to different levels of inflammation.

**Figure 2:**
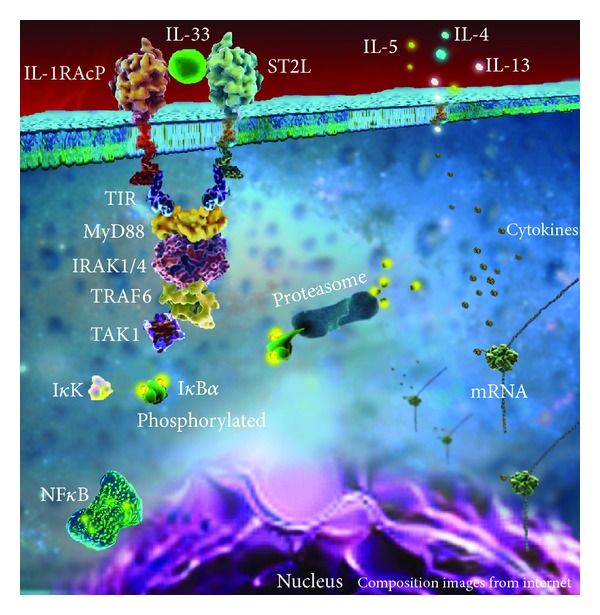
Representation of IL-33 pathway in T-helper cells. IL-33 interacts with ST2L and the receptor accessory protein IL-RAcP in the membrane. Both possess a domain TIR that allows interacting with MyD88, IRAK1/4, TRAF6, and TAK1 in the cytosol. These intracellular signaling molecules determine I*κ*K inactivation by phosphorylation and degradation in proteasome complex. The consequent NF*κ*B activation results in the production of Th2 cytokines.

IL-33 has a single domain that binds to its receptor ST2 in target cells. ST2 (also known as T1, FIT-1, or DER-4) is an IL-1 family receptor, which, as structurally similar receptors IL-1R1 and IL-18R*α*, has three extracellular immunoglobulin-like repeats that belong to the Toll-IL-1 receptor (TIR) super family [23, 34, 35]. ST2 was first identified in 1989 as a serum-inducible secreted protein in fibroblasts and then reported to be regulated by the estrogen-inducible transcription factor Fos [36–38]. It has two splicing variants: sST2 and ST2L. The latter is the long variant, which is fixed to cellular membranes, mainly in Th2 cells and mast cells [39, 40]. The sST2 variant, in contrast, is a soluble form of ST2 that interacts with IL-33 and blocks its biological effects [41]. Importantly, in the IL-33 signaling pathway, ST2L has to be pared with a coreceptor, IL1-RAcP (IL-1 receptor accessory coupled protein), in order to initiate the cascade of signalization [23] (Figure 2).

Contrary to other members of the IL-1 family, IL-33 is more associated with Th2 immune responses. In this regard, the interaction between IL-33 and the complex ST2L/IL1-RAcP induces the recruitment of MyD88, IRAK1/4, and TRAF6 which leads to the activation of NF-*κ*B and Th-2 proinflammatory cytokines, such as IL-4, IL-5, and IL-13 [23, 26]. Accordingly, previous studies have shown that mice treated with an antagonist of ST2 exhibit an enhancement of Th1 response and have an inhibitory effect of Th2 associated allergic airway inflammation [39, 42]. The IL-33/ST2 axis, therefore, has been shown to have an important role in chronic inflammatory conditions associated with a predominant Th2 response. More recently, however, it has been shown that IL-33, although initially labeled as a Th2 cytokine, can also enhance Th1/Th17 immune responses [43–45]. In this regard, IL-33 can induce both Th1 and Th2 responses depending on the stimuli, the cytokine environment, and the cell type involved [44]. It has been shown, for instance, that IL-33 can synergize with IL-1 and IL-18 leading to an enhanced Th1/Th17 response in acute and chronic phases of experimental arthritis [43].

## 2. IL-33 and IBD Animal Models 

In the gut, most data covering the role of IL-33 in intestinal inflammation come from animal studies. In this respect, intraperitoneal injection of IL-33 leads to esophageal inflammation, intestinal goblet cell hypertrophy, and increased production of intestinal mucus in mice, and these animals exhibit infiltration of eosinophils and neutrophils in the colonic mucosa [23]. In addition, it has been shown that intestinal infection with some nematodes in rodents leads to an increase in IL-33 with subsequent upregulation of Th2 cytokines and infection resolution [46]. In IBD, several authors have shown that IL-33 plays an important role in intestinal inflammation using both genetic and chemically induced models. Theresa Pizarro's group, for instance, has shown that IL-33 is increased in mucosa of SAMP1/YitFc mice, which represents a mixed Th1/Th2 model of IBD [16]. Furthermore, several different studies have shown that IL-33 knockout mice are more susceptible to acute dextran sodium sulfate (DSS) administration compared with wild-type animals [47–49]. Those findings were also replicated in the trinitrobenzene-sulfonic-acid- (TNBS-) induced colitis model [20]. In contrast, in the chronic DSS colitis model, weight recovery is markedly delayed in IL-33 knockout mice and the inflammation seems to be less severe when IL-33 is administered to the animals [47]. In mice, the role of IL-33, therefore, seems to be dependent on the stage of inflammation, being detrimental in the acute phase and protective during recovery.

## 3. Lessons from Human Studies

### 3.1. Genetic Evidence

In the past, polymorphisms related to cytokine genes have been shown to be linked to IBD [50–52]. Studies have suggested the association between genetic polymorphisms in the IL-1 family and the development of the disease [52, 53]. With regard to IL-33, Latiano et al. investigated the contribution of IL-33 polymorphisms to the risk of developing IBD, evaluating the existence of possible associations with different disease phenotypes [54]. In a large cohort of adult and pediatric patients, a significant allele and genotype association with IL-33 was found in CD and UC patients. After stratifying for age at diagnosis, differences were still significant only in adult-onset IBD. In addition, an increased frequency of extensive colitis in adults with UC and in steroid-responsive pediatric patients carrying the IL-33 risk polymorphism was observed. In that study, mRNA expression of IL-33 was significantly increased in inflamed IBD biopsy samples. The biologic impact of these polymorphisms, however, is not clear since no differences in IL-33 RNA levels were found when comparing the allele dosage with mRNA expression profiles [54].

### 3.2. Assessment of Human Intestinal Tissue

Between 2010 and 2012, only a few years since IL-33 was first established as a new member of the IL-1 cytokine family, several different groups independently assessed the role of this novel cytokine in IBD using human blood sera and intestinal samples (Table 1). In particular, due to the predominance of a Th2 immune response in UC, several studies have attempted to investigate the role of IL-33 in this specific condition. In previous work, quality of sample description greatly varied among studies. In this regard, a clear description of the sample collection is of most importance since both bowel location and the inflammatory status of the tissue can critically impact results.

Most previous papers, for instance, do not clearly state the exact site of the sample collection in IBD patients and controls. Particularly in the case of CD knowing whether the sample comes from colonic or ileal tissue is critical since these locations greatly differ in histology and biologic function. In contrast, data regarding the inflammatory status of the collected samples are more clearly described. Accordingly, most studies were performed using samples from either involved areas from patients in flare and in remission (healed mucosa) or tissue from involved and noninvolved areas from the same active patient (Table 1). Only one paper evaluating IL-33 in human intestinal mucosa included noninvolved and involved areas from active patients and subjects in remission [20]. In addition to the IBD samples, studies greatly varied with regard to the control group selected, including samples from healthy patients in colon cancer screening, normal looking mucosa of colon cancer patients, irritable bowel syndrome subjects with non-diarrhea phenotype and even controls only vaguely described as “non-IBD” (Table 1). The striking heterogeneity in study design and methods restricts future comparisons among published papers and gives rise to different and occasionally contradictory findings. Most papers, however, seem to point towards the notion that IL-33 is found to be upregulated in inflamed IBD tissue, especially in UC.

### 3.3. IL-33 Is Upregulated in IBD Samples

The main findings with respect to the studies on IL-33 using human samples are described in Table 2. Beltrán et al. showed for the first time that patients with UC had higher IL-33 protein levels in intestinal mucosa compared with CD subjects and healthy controls regardless of disease activity [18]. At RNA level, IL-33 mRNA was also upregulated in UC compared with controls using isolated epithelial cells [17], whole biopsy tissue [15, 16, 19], and surgical specimens [20]. Currently, taking all together, there is enough evidence to state that IL-33 is upregulated in IBD mucosa compared with noninvolved mucosa and controls. Importantly, this increase in IL-33 expression seems to be more prominent in patients with UC.

### 3.4. IL-33 Expression Is Correlated with Disease Activity

It has been further suggested that IL-33 expression is not only upregulated in IBD mucosa, but it also correlates with the inflammatory status. In this regard, Beltrán et al. observed increased levels of IL-33 protein in biopsy extracts of active UC patients compared with patients in remission [18]. Later, Kobori et al. were the first to report an increase in IL-33 expression in mRNA levels in the intestinal mucosa of UC patients with active disease compared with subjects in remission [15]. Of note, the authors showed that the feature was specific for UC as no enhanced expression was found in infectious colitis or CD regardless of inflammatory activity. In keeping with these findings, Pastorelli et al. using whole tissue analysis from affected and nonaffected mucosa of active IBD patients compared with controls observed that IL-33 mRNA transcripts were exclusively more abundant in affected samples from active UC subjects [16]. Seidelin et al. also found increased mRNA levels of IL-33 in active UC compared with patients in remission and controls using RNA from isolated epithelial cells [17]. These results were further confirmed by Sponheim et al. who found elevated levels of IL-33 mRNA in colonic biopsy samples from UC subjects compared with controls and observed that IL-33 values were correlated with clinical activity scores and also with the endoscopic level of inflammation. In addition, Sedhom et al. recently published that, in resection specimens, transcript levels of IL-33 were enhanced within the affected colon mucosa also during remission when compared with noninvolved colonic areas in patients with both UC and CD [20]. Whether patients in remission also display IL-33 upregulation in affected healed mucosa is yet to be confirmed.

### 3.5. IL-33 Is Increased in Sera of IBD Patients

As IL-33 levels in tissue were shown to be correlated with disease activity, many authors have further assessed whether the upregulation of IL-33 in the mucosa of IBD patients could also be reflected by increased levels of the cytokine in sera. In this regard, Beltrán et al. found increased IL-33 levels in the serum of patients with IBD, but no correlation with disease activity was observed [18]. In contrast, Ajduković et al. found no difference in IL-33 serum levels between 18 individuals with UC and healthy controls, suggesting that the role of IL-33 in UC might be posttranscriptional since they could not find any increase in cytokine levels in affected subjects [22]. Later, however, Pastorelli et al. clearly demonstrated that IL-33 serum levels were indeed higher in UC and CD patients compared with controls with no difference between both types of IBD [16]. In this study, only the cleaved form of IL-33 was detectable in humans, suggesting that the cleaved form of IL-33 could serve as a circulating biomarker, particularly in the UC setting. In addition, Pastorelli et al. also showed that anti-TNF therapy could modulate IL-33 serum levels in IBD patients. To evaluate the impact of anti-TNF therapy on IL-33 levels in sera, samples were collected prior to and after infliximab infusions. In the experiment, an acute effect of anti-TNF was detected with a subsequent decline in systemic IL-33 levels. Importantly, circulating IL-33 remained at reduced levels during maintenance therapy, showing that such treatment has long-lasting effects on IL-33 serum levels [16]. Nevertheless, the clinical and prognostic consequence of the aforementioned effect remains to be established by larger cohort studies.

### 3.6. *In Situ* IL-33 Expression in Human Intestine

The *in situ* expression of IL-33 in intestinal mucosa has been investigated in great part by immunohistochemistry and immunofluorescence techniques. In an approach based on immunofluorescence, for example, IL-33 was shown to be predominantly expressed in intestinal epithelial cells of patients with IBD and controls [18]. In healthy and CD intestinal mucosa, IL-33 seems to localize within the cytoplasm of epithelial cells; whereas in UC patients IL-33 expression was suggested to be decreased and an enhanced nuclear staining was detected [18]. Later, Seidelin et al. confirmed by immunohistochemistry that IL-33 was expressed in epithelial cells of UC patients; no staining was detected in control specimens [17]. Kobori et al, however, observed no IL-33 staining in intestinal epithelial cells, but, instead, expressing cells coincided with a-SMA-positive cells located in the subepithelial regions, suggesting that human colonic subepithelial myofibroblasts could represent a major source of mucosal IL-33 [15]. Similar results were also observed by Sponheim et al. in surgical specimens [19]. In that study, the nuclear expression of IL-33 was not found in intestinal epithelial cells from mucosal samples of healthy controls while seldom detected in patients with IBD. Instead, the authors suggested that cells with IL-33-positive nuclei (myofibroblasts) in ulcerations of UC samples were accountable for the IL-33 expression [19]. Others, however, have confirmed that there is both epithelial and subepithelial expression of IL-33 in intestinal mucosa, with predominance in epithelial cells [16–18, 20]. Variations in IL-33 expression could be explained by the assessment of different samples (biopsy or resection specimen) and the use of different antibodies or staining methods. In this regard, Sponheim et al. suggested that the fact that they evaluated larger samples from bowel resections enabled the discovery of an enhanced IL-33 signal in ulcerations, a finding that could have been missed in smaller samples [19].

### 3.7. ST2 Expression in Human Intestine

The main findings with respect to ST2 in human samples can be found in Table 3. Beltrán et al. showed for the first time that ST2 was upregulated in mucosa of patients with IBD, with ST2 expression being higher in UC compared with CD and controls [18]. The soluble form of ST2 might be responsible for these results as it was shown to be upregulated in both protein and mRNA levels in patients with UC. In keeping with these findings, Pastorelli et al. evaluating ST2 expression in biopsy samples and resection specimens found that the soluble form of ST2 was indeed increased in active IBD, particularly in UC [16]. With respect to ST2 expression *in situ*, Beltrán et al. observed a loss of ST2 staining in the intestinal epithelium of UC patients; the staining was limited to the lamina propria, expressed by infiltrating macrophages and lymphocytes [18]. In inflamed UC, Sedhom et al. also showed that subepithelial infiltrates had many cells positive for ST2 in active and nonactive IBD [20]. In contrast, in controls, ST2 was expressed by the epithelium, suggesting that there is indeed an epithelial loss of the membrane-anchored long form of ST2 during inflammation. Taken together, these results suggest that in active IBD there is loss of the membrane-bound long form of ST2 in the epithelium with subsequent increased expression of the soluble form. This fact seems to be specifically related to IBD because, in contrast, ST2 appears to be upregulated in both epithelium and lamina propria in patients with infectious colitis and diverticulitis [16].

### 3.8. The IL-33/ST2 Axis

As the soluble form of ST2 has been shown to act as a decoy receptor, the aforementioned findings suggest that, during mucosal inflammation, there may be an ST2 related autoregulation of the pathway by loss of the membrane-bound long form of ST2 and a shift towards the soluble form in the epithelium. Furthermore, the long form of ST2 seems to be the predominant isotype expressed in the epithelium, which is lost during active UC with an increased presence of the soluble isoform. The different IL-33 isoforms also play a role in this autoregulation as it has been shown that cell death associated proteolysis by caspases 3 and 7 can downregulate the proinflammatory properties of IL-33, cleaving the cytokine into its less active forms [30]. At the same time, the proinflammatory microenvironment can also potentially amplify the function of IL-33 by the release of elastase and cathepsin G by neutrophils giving rise to a lighter form of IL-33 with enhanced biologic properties [32].

### 3.9. Future Perspectives

IL-33 is ubiquitously expressed in different cells and has multiple biological functions, ranging from the regulation of epithelial homeostasis to the orchestration of the Th2 type of immune response. Concerning the gastrointestinal tract, IL-33 expression has been independently investigated in distinct inflammatory disorders. In human IBD, especially in UC, the IL-33 overexpression may reflect and further support the presence of subtle abnormalities of the innate immunity underlying IBD pathogenesis. Whether the abnormal expression or dynamic changes of IL-33 represent a primary defect or a secondary phenomenon in the IBD pathogenesis remains to be established. Further studies will be necessary in order to thoroughly investigate the exact role of IL-33 in human IBD and other chronic inflammatory diseases involving the gastrointestinal tract.

## Figures and Tables

**Table 1:** Studies evaluating IL-33 in intestinal samples from inflammatory bowel disease patients are listed chronologically with data regarding the sample collection site and the control group.

Studies	IBD patients in remission		IBD patients in flare		Controls
	Noninvolved area	Involved area (healed)	Noninvolved area	Involved area	
Kobori et al., (2010) [15]			*✓*	*✓*	Cancer patients
Pastorelli et al., (2010) [16]			*✓*	*✓*	Cancer screening
					Diverticulitis
					Infectious colitis
Seidelin et al., (2010) [17]		*✓*		*✓*	Cancer screening
Beltrán et al., (2010) [18]		*✓*		*✓*	Non-IBD
Sponheim et al., (2010) [19]		*✓*		*✓*	Irritable bowel syndrome
Sedhom et al., (2012) [20]		*✓*	*✓*	*✓*	Cancer patients
					Cancer screening
Wakahara et al., (2012) [21]			*✓*	*✓*	Non-IBD

**Table 2:** Studies covering the role of IL-33 in inflammatory bowel diseases using human samples are listed chronologically with data regarding the assessed disease, method of analysis, and main results.

Studies	Disease	Sample	Method	Results	Localization
Ajduković et al., [22] (2010)	UC	Serum	ELISA	IL-33 not increased compared to controls	NA

Kobori et al., [15] (2010)	UC CD	Colonic biopsies	qPCR IHC	↑IL-33 in active UC	Subepithelial myofibroblasts

Pastorelli et al., [16] (2010)	UC CD	Colonic biopsies	qPCR WB	↑IL-33 in active IBD (UC > CD)	NA
		Surgical specimens	IHC	↑IL-33 in active UC	Intense staining mainly localized to the epithelium and infiltrating LPMC
		IEC isolated from surgical specimens	qPCR WB	↑IL-33 in active UC	IL-33 is predominantly expressed by IEC in active UC
		Serum	ELISA WB	↑IL-33 in active IBD Only cleaved IL-33 in sera	NA

Seidelin el al., [17] (2010)	UC	IEC isolated from biopsies	qPCR WB	↑IL-33 in active > inactive > controls	Localized in the epithelium and infiltrating lymphocytes

Beltrán et al., [18] (2010)	UC CD	Serum	ELISA	↑IL-33 in IBD patients	NA
		Colonic biopsies	ELISA IF	↑IL-33 in active IBD	In controls and CD, IL-33 was localized in the cytoplasm of epithelial cells. In UC, a decreased cytoplasm staining was observed. Both IBD showed strong nuclear staining

Sponheim et al., [19] (2010)	UC CD	Colonic biopsies	qPCR	↑IL-33 in UC	NA
		Surgical specimens	IHC	Nuclear expression was seen only rarely in crypts of IBD samples	In UC, focal accumulation of cells with IL-33-positive nuclei underlying ulcerations was found (myofibroblasts)

Sedhom et al., [20] (2012)	UC CD	Surgical specimens	qRT-PCR ELISA IHC WB	↑IL-33 in active colonic tissue versus noninvolved areas	In involved mucosa, nuclear IL-33 was found in colonic epithelial cells. In CD, inflammatory aggregates were found surrounding IL-33+ cells. In UC, IL-33+ cells formed “shield-like” clusters in ulcers

Wakahara et al., [21] (2012)	UC CD	Colonic explant culture	ELISA	↑IL-33 in IBD patients. No difference between active versus inactive sites	NA

qRT-PCR: quantitative polymerase chain reaction, IF: immunofluorescence, IHC: immunohistochemistry, WB: western blot, LPMC: lamina propria mononuclear cells, IEC: intestinal epithelial cells, (↑) increase, and (NA) nonapplicable.

**Table 3:** Studies evaluating the IL-33 receptor ST2 in inflammatory bowel diseases using human samples are listed chronologically with data regarding the assessed disease, method of analysis, and main results.

ST2	Disease	Sample	Method	Results	Localization
Pastorelli et al., [16] (2010)	UC CD	Colonic biopsies	qPCR WB	↑Total ST2 mRNA levels were observed in active UC with specific abundance of sST2. No significant changes were detected for ST2L	NA
		Surgical specimens	IHC	↑ST2 staining was observed in inflamed UC. ↓Intense but similar pattern was observed in CD.	In inflamed UC, ST2 was limited to the LP in infiltrating macrophages and lymphocytes. In controls, the primary source for ST2 was the epithelium
		IEC isolated from surgical specimens	qPCR WB	↓Total ST2 mRNA in UC versus controls while significant variability was found in CD. ↑ST2L in controls. ↑sST2 in IBD in general.	Epithelial loss of ST2 during inflammation is characteristic of IBD due to a decrease in ST2L.
		Serum	ELISA	↑circulating sST2 levels were found in both UC and CD versus controls	NA

Beltrán et al., [18] (2010)	UC CD	Serum	ELISA WB	↑ST2 in IBD versus controls. Active UC > remission	NA
		Colonic biopsies	ELISA WB IF	↑ST2s mRNA was observed in active UC versus CD and controls. In WB, ST2s was only detected in UC. ST2s/ST2L expression was ↑ in active UC. ↑ST2 is due to ↑ST2s expression	Observed loss of ST2 staining in the epithelium in UC patients with strong expression observed in the cytoplasm and in the apical surface of crypt epithelial cells

Sedhom et al., [20] (2012)	UC CD	Surgical specimens	IHC	ST2 staining was found in the mucosa of UC and CD and in controls	ST2 is expressed by colonocytes and its expression is barely detectable among leukocytes in the lamina propria. Subepithelial infiltrates contained many ST2-positive cells in either active or nonactive IBD

qRT-PCR: quantitative polymerase chain reaction, IF: immunofluorescence, IHC: immunohistochemistry, WB: western blot, LPMC: lamina propria mononuclear cells, IEC: intestinal epithelial cells, (↑) increase, (↓) decrease, and (NA) nonapplicable.
